# Facilitators and barriers to modifying dietary and hygiene behaviours as adjuvant treatment in patients with depression in primary care: a qualitative study

**DOI:** 10.1186/s12888-018-1779-7

**Published:** 2018-06-19

**Authors:** B. Olivan-Blázquez, J. Montero-Marin, M. García-Toro, E. Vicens-Pons, M. J. Serrano-Ripoll, A. Castro-Gracia, M. C. Sarasa-Bosque, J. M. Mendive-Arbeloa, Y. López-del-Hoyo, J. Garcia-Campayo

**Affiliations:** 1 Department of Psychology and Sociology, University of Zaragoza, Violante de Hungria 23, 50009 Zaragoza, Spain; 2 Research Network on Preventive Activities and Health Promotion (Red de Investigación en Actividades Preventivas y Promoción de la Salud - RedIAPP), Barcelona, Spain; 3 Promosam Network, Red de Excelencia PSI2014-56303-REDT PROMOSAM: (Investigación en procesos, mecanismos y tratamientos psicológicos para la promoción de la salud mental), Economy and Competitiveness Ministry, Madrid, Spain; 4 Institut Universitari d’Investigació en Ciències de la Salut (IUNICS), Universitat de les Illes Balears (UIB), Palma de Mallorca, Spain; 5 Departamento de Psiquiatría, Parc Sanitari San Joan de Déu, Sant Boi de Llobregat, Spain; 6 Aragones Health Service, Zaragoza, Spain; 7 Institut Català de la Salut, Barcelona, Spain; 8 Department of Medicine and Psychiatry, University of Zaragoza, Zaragoza, Spain

**Keywords:** Depression, Lifestyle, Intervention programmes, Facilitators, Barriers

## Abstract

**Background:**

Major depression is a highly prevalent condition. Its pathogenesis is related to a wide variety of biological and psychosocial factors and among these is factors related to lifestyle. Lifestyle-based interventions seem to be appropriate strategies as coadjutant treatment. The objective of this study is to explore and identify expectations and experiences of both patients and healthcare professionals that can point to the main barriers and facilitators with regard to the promotion of healthy dietary and hygiene behaviours in patients suffering from major depression.

**Methods:**

A qualitative design was used to collect information from a wide range of purposefully and theoretically guided samples of depressed patients and health professionals from Primary Care (PC). Both in-depth interviews and discussion groups were used. A standardized protocol was designed to guide the interviews and groups, including the preparation of a topic list to be addressed, with previously tested, open suggestions that could be of interest. A thematic analysis was performed from grounded theory in order to explore, develop and define until saturation the emergent categories of analysis derived from the individual interview and group data.

**Results:**

Both patients as well as PC professionals noted a series of central aspects with respect to the implementation of a programme for the acquisition of healthy dietary and hygiene habits for depressive patients, which may be organized around ‘personal’, ‘programmatic’, and ‘transversal’ aspects. As for the personal aspects, categories regarding ‘patient history’, and ‘disposition’ were found; the programmatic aspects included categories such as ‘presentation and monitoring’, and modification of ‘cognitive’ and ‘behavioural’ habits; whereas the transversal aspects comprised the possibilities of ‘social support’ and defining categories of ‘objectives’.

**Conclusion:**

The implementation of intervention programmes that combine dietary and hygiene-related factors in patients with depression is complex, given the nature of the disorder itself, and its symptoms such as apathy and feelings of guilt or incompetence*.* Key issues exist for the success of the intervention, such as the simplicity of guidelines, tailoring through motivational interviewing, prolonged and intense monitoring throughout the different stages of the disorder, and the provision of adequate feedback and social support. PC could be an appropriate level in which to implement these interventions.

## Background

Major depression Disorder (MDD) is a highly prevalent condition and has become the second most common cause of disease-induced disability in our society [1–3]. Its pathogenesis is related to a wide variety of biological and psychosocial factors. Among these are factors related to lifestyle, as diverse studies claim [4–9].

The relationship between depression, diet and obesity is well established [10], as is that between eating style, weight gain and depression [11]. The comorbidity of obesity and depression is highly significant, with depression being up to 20–45% more prevalent in obese individuals [12]. A systematic review of prospective studies describes a reciprocal cause-effect relationship between depression and obesity [12]. Furthermore, weight loss in the obese has been found to reduce symptoms of depression [13], and similarly an improvement in short-term depression has been associated with weight loss, not only in females [14], but also in the general population [15]. A poor diet, obesity and/or metabolic syndrome predispose individuals to metabolic changes that interact with cerebral function through very complex mechanisms, probably having an individualized effect on each patient [10, 16–18]. The alteration of certain metabolic mechanisms has been associated with a poor outcome for depression, but this phenomenon also occurs in the opposite direction, creating a vicious circle of illness [10, 19]. Lee et al. [20] highlight the complex relationship between antidepressant drugs, MDD and weight gain. Hypothalamic-pituitary-adrenal (HPA) axis activation occurs during states of stress; concurrently, the HPA axis is also dysregulated in obesity and metabolic syndrome, making it the best understood shared common pathophysiological pathway with MDD.

Given this close relationship, lifestyle – more specifically dietary and hygiene behaviours – could play a major role in the initiation, maintenance and treatment of depression, at least as an adjuvant [4, 7, 19, 21]. The combination of different lifestyle-based interventions (diet, exercise, sun exposure, and sleep hygiene) appears to be an appropriate strategy to increase their effectiveness [19, 22], although it has been poorly investigated and has had varied results [4, 19, 23]. Furthermore, no studies exist regarding the difficulties and opportunities for change and how the healthcare system could help patients suffering from depression to modify their dietary and hygiene behaviours, as compared to other medical conditions such as diabetes [24, 25], Crohn’s disease [26] or cardiovascular disease [27].

Primary Care (PC) is the most accessible healthcare service, offering comprehensive and continuous patient-centred care, and it is the most frequently used by citizens. Therefore, it is an ideal scenario for conducting individual, group and community interventions to change behaviours [28]; in this case, those related to the treatment of depression [19] or even its prevention [29].

Thus, the objective of this study is to explore and identify facilitators and barriers to the promotion of healthy dietary and hygiene habits in patients suffering from major depression from the expectations and experiences of both patients and healthcare professionals. This study was developed in PC centres and those attending to the most important aspects when implementing behaviour modification programmes in this population.

## Methods

A qualitative design was used to collect information from a wide range of purposefully and theoretically guided samples of depressed patients and health professionals from PC. With the intention of increasing the consistence of the study, both in-depth interviews (with depressed patients) and discussion groups (with depressed patients and health professionals, separately) were used to access the subjectivity and the processes involved in generating ideas and concepts [30]. This triangulation of techniques and informants has been used in other works [31], with the assumption that qualitative research is inherently multi-method [32]. In-depth interviews were conducted by a single interviewer, and discussion groups were moderated by an interviewer and an observer, both of them female psychologists and researchers with previous experience in the field and no previous contact with participants.

Patients were recruited from the Spanish autonomous regions of Aragon and the Balearic Islands during their visits to PC, taking advantage of their participation in a research project (randomized controlled trial, RCT) on lifestyle change recommendations in patients with major depression [10, 23, 33]. This RCT consisted of a series of written dietary and hygiene-related lifestyle recommendations to improve their depressive symptoms and quality of life with low socio-sanitary costs. Through a randomized, double-blinded, multicentre, two arm-parallel clinical trial, with 12 month follow-up, and a sample of 273 PC patients, it was observed that just giving written lifestyle recommendations is not enough for depressive patients to benefit from them. Therefore, there seems to be a need for deeper understanding into the patient and professional expectations and experiences that might point to the barriers and facilitators to the implementation of these kinds of programmes. For this purpose, a subsample of patients was created, selected after stratification based on the variables of age, (18–40; 41–60; > 60), sex, level of education (primary, secondary, tertiary), occupation (paid, unpaid, no occupation) and level of depression at the basal assessement of the RCT using the Beck depression inventory [34] (mild, moderate, severe), treatment group in the study (active vs control), and city (Palma, Zaragoza). Health professionals were contacted by telephone, based on their age (18–40; 41–60; > 60), sex, occupation (general practitioner vs nurse), years of experience (< 15; 15–30; > 30), type of work contract (temporary vs permanent), participation in the study (participation vs not participation) and city (Palma, Zaragoza), in order to gather plentiful and varied information. Both patients and professionals were aware of the research project about lifestyle change recommendations, but none of them had any knowledge on the group to which patients were assigned. Every contacted patient and health professional initially agreed to be interviewed, but a number of them eventually did not keep the appointment owing to incompatible schedules. Table 1 outlines the main characteristics of the final 41 participants (11 patients and 30 professionals). The profile of a participating patient is a woman, between 40 and 60 years of age, with primary studies and mild or moderate depression. The profile of a participating health professional is a woman, between 40 and 60 years of age, general practitioner, with a permanent contract and more than 15 years of professional experience. The interviews were conducted between November 2014 and January 2015.

**Table 1:** Characterístics of patients and health care professionals

Variables	Patients (*n* = 11)
Age	
20–40 years	1 (9.1%)
41–60 years	6 (54.5%)
> 60 years	4 (36.4%)
Sex	
Male	3 (27.3%)
Female	8 (72.7%)
Education	
Primary	6 (54.5%)
Secondary	4 (36.5%)
Tertiary	1 (9.1%)
Occupation	
Paid	2 (18.2%)
Unpaid	4 (36.4%)
No occupation	5 (45.5%)
Level of depression	
Mild	4 (36.4%)
Moderate	4 (36.4%)
Severe	3 (27.3%)
Group	
Active	6 (54.5%)
Control	5 (45.5%)
Variables	Professionals (*n* = 30)
Age	
20–40 years	8 (26.6%)
41–60 years	17 (56.7%)
> 60 years	5 (16.7%)
Sex	
Male	11 (36.7%)
Female	19 (63.3%)
Occupation	
General practitioner	23 (76.7%)
Nurse	7 (23.3%)
Experience	
< 15 years	9 (30%)
15–30 years	10 (33.3%)
> 30 years	11 (36.7%)
Type of work contract	
Temporary	10 (33.3%)
Permanent	20 (66.7%)
Group	
Participation	16 (53.3%)
No participation	14 (46.7%)
City	
Zaragoza	26 (86.7%)
Palma	4 (13.3%)

A standardized protocol was designed to guide the interviews and groups, including the preparation of a topic list to be addressed, with previously tested, open suggestions that could be of interest. The topic list was compiled from the experience of a panel of expert researchers who had developed and conducted a previous study on hygienic-dietary recommendations as adjuvant treatment in depression [19, 35] and taking into account the NICE behaviour change recommendations [36]. This topic list, shown in Table 2, comprised the following: previous knowledge of lifestyle recommendations (diet, physical exercice, sun exposure, sleep hygiene) as an adjuvant treatment for depression; where there was compliance with instructions: difficulties, feelings; in case of non-compliance with recommendations: causes, feelings; perception of efficacy; aspects that facilitate compliance with recommendations; appropriate level or means to change behaviour; ability of health system to help patients to change their behaviour and mood; proposals by health professionals when consulted about a mood problem; type of proposals that health professionals give and their characteristics; type of proposals that can be given; effectiveness of the proposals. After a short introduction about the study, patients and health professionals were asked open, general questions in order to raise a response and begin discussion. More direct questions were asked when specific topics did not spontaneously arise in the discourse. These are given in Table 3.

**Table 2:** Topic list

- Previous knowledge of lifestyle recommendations (diet, physical exercise, sun exposure, sleep hygiene) as an adjuvant treatment for depression. - Compliance with instructions. - Difficulties - Feelings - In case of non-compliance with recommendations: causes, feelings. - Perception of efficacy - Aspects that facilitate compliance with recommendations - Appropriate level or means to change behaviour. - Ability of healthcare system to help patients change their behaviour and mood. - Proposals by health professionals when consulted about a mood problem - Type of proposals that health professionals offer and their characteristics. - Type of proposals that can be made. - Effectiveness of the proposals

**Table 3:** Questions asked to patients and healthcare professionals

Patients	Professionals
- Do you know anything about lifestyle modification (diet, exercise, etc.) in order to change your mood and depression? - All of you have taken part in a research project about modifying lifestyles. Did these recommendations that were given to you show you something new that you did not know? - Did you follow the recommendations for modifying lifestyles? If NOT, why didn’t you follow them? - How did you feel when you followed the recommendations? And when you didn’t? - What difficulties did you encounter in implementing the recommendations? - Do you think these recommendations helped you? - What do you think about the doctor’s role? - And about the way of carrying out this modification? - Do you think another type of intervention would have helped you? Would an educational group or another kind of intervention have helped you? - Do you think there is something more that can be done to help you to modify your lifestyle when depressed?	- How do you think you should make recommendations for modifying life styles? - And to a patient with depression? - Do you know how this intervention can help him/her? - Do you think these recommendations can be effective? And do you believe the patient thinks that they work? - What difficulties do you think depressed patients have in changing their behaviour? - And what feelings do they have? - And as for modifying lifestyles, do you work in a different way in the case of a depressed patient? - What do you think is necessary in order to be effective when modifying lifestyles? - What do you think about educational groups? - And about follow-up? - Do you think these interventions are feasible from PC? - What difficulties do you encounter when giving this type of advice? - What is the appropriate level (or department or system) to implement this kind of interventions?

The objectives of the study were indirectly raised and questions asked about the topics in an open and progressive way. The interviewers and/or moderators were introduced to the participants as research psychologists and assumed a minimally orientative role, limiting their interventions to addressing the topics in the script. The settings for data collection were a neutral room in the different health centres at which the patients were registered, without the presence of non-participants. In-depth interviews lasted between 35 and 55 min and the discussion groups lasted 50–75 min. All of them were digitally audio-recorded and a verbatim transcription made in order to obtain the final set of qualitative data for the analysis, which was revised by the participants and added to the field notes made during and after the interviews/groups. An inductive thematic content analysis was performed from grounded theory in order to explore, develop and define the emergent categories of analysis derived from the individual interview and group data until saturation [37–40].

The type of analysis used was therefore of a qualitative, vertical and interpretative nature, with stratified and projected sampling aimed at gathering plentiful and varied information, and with an emerging and non-frequential design for the analysis categories [41, 42]. The aplication of the ‘constant comparative method’ strategy enabled conceptual categories to be systematically generated, and analysis and explicit coding to be combined with theory building, highlighting significant properties and relationships among all of them [43]. Specifically, we used ‘open coding’ to develop the first provisional interpretations; ‘axial coding’ to deepen and discover properties and relationships in each category; and also a ‘core category’, which was able to integrate and summarize all the emergent categories by means of ‘selective coding’ that finally provided the esential framework [44, 45]. Theory integration throughout the design, implementation and evaluation of the previously mentioned parallel RCT intervention also provided valuable insight into how these emergent categories contribute to effectiveness in changing behavioral outcomes [46].

All the analyses were performed iteratively using Maxqda-2007 software in agreement between two researchers (BOB, JMM), and the interpretations made from the data were discussed with interviewers (PR, PHM) and participants to obtain their approval [47]. This methodological triangulation was able to increase consistency and rigour by combining multiple techniques and maximizing the breadth and depth of perspectives.

Participants provided written informed consent to participate in the study. No interview/group was repeated. As previously explained, this study forms part of a research project that includes an RCT on the effectiveness of a lifestyle change recommendation programme in depressed patients [10, 23, 33]. Once the clinical trial stage was completed, this qualitative study was carried out to analyse the facilitators and barriers that patients with depression experience with regard to modifying their dietary and hygiene behaviours. The project was independently approved by the Research Ethics Committee of Aragon (PI12/0022) and the Research Ethics Committee of the Balearic Islands (PI11/1563), Spain, on March 2012.

## Results

**Fig. 1:**
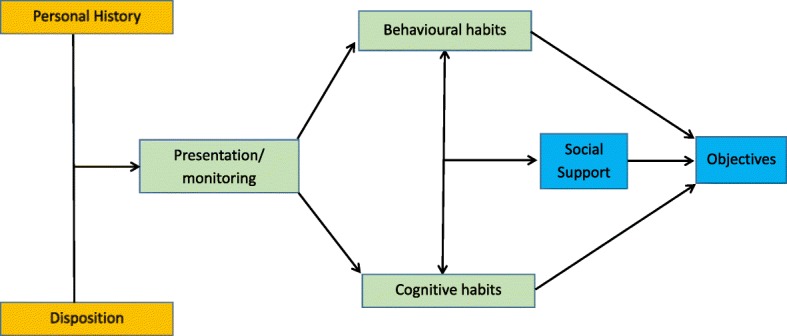
Categories for the implementation of a programme for the acquisition of healthy dietary and hygiene habits in patients with major depression. Note: ‘Personal’ aspects are in orange. ‘Programmatic’ aspects are in green. ‘Transversal’ aspects are in blue

Both patients and PC professionals noted a series of central aspects with respect to the implementation of a programme for depressive patients to acquire healthy dietary and hygiene habits, which may be organized around ‘personal’, ‘programmatic’, and ‘transversal’ aspects. In relation to personal aspects, from the patients’ perspective, topics regarding their ‘personal history’ and their personal readiness or ‘disposition’ were found. On the other hand, the programmatic aspects included topics such as programme ‘presentation and monitoring’, from both the patients and professionals’ perspective; the modification of ‘cognitive habits’, from patients’ perspective; and the modification of ‘behavioural habits’, from professional’s point of view. All of them were interconnected in a synergetic manner towards the achievement of the transversal aspects, which were delimited by the possibilities of ‘social support’, from patients’ perspective, and definition of ‘objectives’, from professional’s point of view. Figure 1 provides a graphic representation of the core aspects that configure the central category (e.g., personal, programmatic, and transversal), and the main categories for the implementation programme for patients with major depression to acquire healthy dietary and hygiene habits. Table 4 provides quotes (Q) in which the properties or characteristics that make up each of the emerged categories are explained in the participants’ own words.

**Table 4:** Quotes regarding the results and relationships with emergent categories

Core aspects	Categories	Properties	Quotes
Personal aspects	Personal History (patient’s perspective)	-History and comorbidity	*Q1: “While my husband was ill [with cancer], I didn’t cry… But when he died, my life changed completely…. I was not the same person…, I withdrew into myself…”* (Patient 7) *Q2: “I have osteoarthritis... and chronic lower back pain… a lot of pain”.* (Patient 6)
		-Vital changes	*Q3:“When you have a problem, if you don’t change the way you live, you don’t solve it”. (Patient 9)*
	Disposition (patient’s perspective)	-General facilitators (e.g. simplicity)	*Q4: “…very simple and effective things… very easy to do things, and they don’t involve anything out of the ordinary”.* (Patient 3)
		-General barriers (e.g. apathy)	*Q5:“You read the recommendations several times and yes, you see that they’re good… but you aren’t going to follow them every day. You are stuck and wound up and you can’t pick yourself up... There are always things that make you lose heart”.* (Patient 4)
Program aspects	Presentation and monitoring	-Instructions (patient’s perspective)	*Q6: “I would have appreciated receiving more information and more encouragement”.* (Patient 1)
		-Assessment (professional’s perspective)	*Q7: “The degree of compliance has to be objectified and measured in some way in order to obtain clear conclusions about the intervention and the change in mood”.* (General practitioner 8)
		-Supervision (patient’s perspective)	*Q8: “The fact that the psychologist would phone me and ask me how I was doing and about whether I was following instructions was a big help for me”.* (Patient 8)
		-Pharmaceutical consumption (professional’s perspective)	*Q9: “You have to explain what the drugs are, the possible side effects, how long it will take to feel an effect and how long the treatment can last”.* (General practitioner 25)
	Cognitive habits (patient’s perspective)	-Agency	*Q10: “Motivation, will, determination. It costs a lot of work and you go as you can, but you end up improving”.* (Patient 11)
		-Ruminations	*Q11: “You feel useless, absolutely useless… why should you do anything if there’s no point? I don’t think that any more, but at the time I couldn’t help but feel that way.”* (Patient 9)
		-Avoidance	*Q12: “The only thing that saved me was reading; it blocked everything out and I found some relief.”* (Patient 3)
		-Restructurings	*Q13: “I see that when other people don’t feel like doing something, they don’t, and nothing happens. Why should it be different for me? Well, I also have to get on with things my way.”* (Patient 4)
		-Guilt	*Q14: -So you are blaming yourself for failing…* *-Yes, that’s right.* *-Guilt is the first problem in this illness…* *-Guilt is very difficult…* *-It’s a very important factor…* (Interactions of several patients in a focus group)
	Behavioural habits (professional’s perspective)	-Activation	*Q15: “We have to motivate our patients to go out and stay in touch with people, but gradually, with a guide, for instance by avoiding situations of conflict at the beginning, because they have to make a great effort at this stage”.* (General practitioner 21)
		-Empowerment	*Q16:“When dealing with a person suffering from depression, we’re starting with a very low level of motivation”. (*General practitioner 12) *Q17:“A person with depression has their ups and downs; if you want your advice to have an effect, you have to wait until the right moment, when the person is open to it”.* (General practitioner 18)
		-Time pressure	Q18:*“…high-speed medicine, which means you have to get eight messages across to them in five minutes”.* (General practitioner 18)
		-Habit control	*Q19:“There’s no doubt about it that if you’re going to change your eating habits, you must have control over your diet. You have to do it yourself, because if you eat out for work or if somebody prepares your meals for you, it’s more complicated”.* (General practitioner 5)
		-Opportunities	*Q20:“One difficulty that patients may find when changing their habits, especially with regard to food, is cost... eating certain foods is more expensive than eating others”. (General practitioner 8)* *Q21: “Patients should be able to go for a walk or do exercise in nice places, in a pleasant urban environment that is well looked after”.* (General practitioner 23)
Transversal aspects	Social Support (patient’s perspective)	-Group format of intervention	*Q22:“The (intervention) group helped a lot because you realize that there are other people who are in the same situation as you.”* (Patient 5)
		-Contact with others	*Q23: “Having contact with others has been very useful for me. Going out for coffee with someone, people phoning me and showing interest in me, regaining face-to-face contact”.* (Patient 8) *Q24:“When you’re depressed, you need somebody to help you along, and your family and friends are there for that. But when you’re really down, your family can lose patience, friends too; so it’s important to have someone from outside to lend you a hand.” (Patient 2)* *Q25:“The group is a good thing, but then you need to have your personal, more private space….”* (Patient 8)
	Objectives (professional’s perspective)	-Adjustment	*Q26:“…because you can’t ask people to do something they’ve never done before or something that is very difficult for them”.* (General practitioner 11). *Q27: “I believe that this programme would obtain better results by applying it to prevent recurrences in patients who have already improved their depression (...) that is, to acquire this lifestyle and maintain it.”*(General practitioner 21).
		-Intervention level	*Q28: “When it comes to personal level intervention, healthcare professionals should be the ones to do it, but if we’re talking about a mass intervention, the media – radio, television, press – should be involved.”* (General practitioner 15) Q29: “*…in schools for young children… I think schools should teach children healthy lifestyle habits from a very young age*”. (General practitioner 17)

### Conceptualization of the discourse

#### Personal aspects

Personal aspects emerged from the patients’ discourse, revolving around their ‘personal history’, including subtopics, such as ‘history and comorbidity’, in relation to patient background and development (Q1), and with reference to comorbidities with other physical illnesses that prevent behaviour modification (Q2). Within the patients’ ‘personal history’, ‘life changes’ were also noted, whereby lifestyle modifications would have significant effects on the course of the illness (Q3). Personal ‘disposition’ also appeared, with subtopics that function as general ‘facilitators’, such as the simplicity of instructions (Q4), as well as general ‘barriers’, including aspects that hinder participation in and commitment to the programme, such as apathy (Q5), one of the symptoms of the illness.

#### Programmatic aspects

Programmatic aspects included topics such as programme ‘presentation and monitoring’ or follow up, which from the patient’s perspective meant receiving appropriate ‘instructions’ on the quantity of information and stimuli (Q6). However, from a professional perspective, it took on the form of a suitable ‘assessment’, as reflected in its interest as a form of objectifying the level of programme adherence and change in mood (Q7). On the other hand, programme ‘presentation and monitoring’ for patients also included the idea of ‘supervision’ throughout the intervention process (Q8). However, for the professionals, the idea of also explaining the pharmacological treatment, and how habit modification could improve it, was found to be more relevant. The appropriate monitoring or follow-up of ‘pharmaceutical’ consumption would be essential from their point of view (Q9). Programmatic aspects also included the patients’ handling of ‘cognitive habits’, such as the idea of ‘agency’ in the sense of determination and motivation (Q10) or ‘ruminations’, which are negative thoughts that hinder the implementation of a new, healthier life style (Q11). Other subtopics were found within the ‘cognitive habits’ category, such as ‘avoidance’, considered to be cognitive avoidance of responsibilities (Q12), as well as the cognitive ‘restructurings’ that take place throughout the new lifestyle implementation process (Q13). Finally, the differences that lead to the implementation of new lifestyle based on dietary and hygiene behaviours generated a certain sense of ‘guilt’ in patients (Q14). Programmatic aspects for the professionals also included the handling of ‘behavioural habits’, including the idea of behaviour ‘activation’ (Q15) and patient ‘empowerment’ (Q16, Q17), and also noting the ‘time pressure’ as a difficulty in carrying out patient care at this level (Q18). Other subtopics were also included such as ‘habit control’ (Q19) and the influence of the acquisitive power or ‘opportunities’ (Q20, Q21).

#### Transversal aspects

Transversal aspects emerged as an area dependent on all the above properties, which included the possibilities of ‘social support’ and defining the ‘objectives’ categories. The ‘social support’ category emerged from the patients’ discourse and included the social possibilities of the ‘group format’ interventions, which are well accepted (Q22), although without losing individual ‘contact with others’, especially face-to-face contact beyond family members and friends, in order to gain a more private support space (Q23, Q24, Q25). Finally, the category of defining ‘objectives’ appeared as an assessment that was also dependent on all of the above, when considering the difficulty of implementing a programme of this sort. According to the professionals, the need for ‘adjustment’ to the specifics of each patient property included – the notion that this type of programme could perhaps better improve the prevention of recurrences in patients who have already improved (Q26, Q27). The ‘intervention level’ property showed that the programme should be implemented at PC level –in coordination with the general practitioner and nurse – while also noting the advertising campaigns that have a great influence on the patients and their decision to follow specific lifestyle patterns (Q28), and the importance of education – beginning at primary level – on the acquisition of healthy lifestyle habits (Q29).

Table 5 shows the theoretical definitions for the basic properties of the model based on the results, summarizing the main influencing factors that both patients and healthcare professionals highlighted, in order to change behaviours of depressed patients, with especial emphasis on modifications to dietary and hygiene behaviour in order to adopt a healthier lifestyle.

**Table 5:** Theoretical definitions for the properties of the model

Core aspects	Categories/Properties	Definitions
Personal aspects	Personal history	
	-History and comorbidity	History, aetiology, course, severity of depressive disorder and comorbidity that determines psychological capacity.
	-Vital changes	Modifications in forms and lifestyles with significant effects for the subject.
	Disposition	
	-G. Facilitators	Personal or environmental aspects that encourage participation and engagement with the programme, like the feeling of improvement, the practice of certain activities, or the ease and simplicity of compliance.
	-G. Barriers	Personal or environmental aspects that complicate participation and commitment to the programme, such as the symptoms of the actual disorder, and apathy.
Program aspects	Presentation and monitoring	
	-Instructions	Clear presentation and explanation of the recommendations that make up the programme, using different channels and giving them the importance that they deserve, but with some flexibility, progressiveness and tailoring so that they are possible to implement.
	-Assessment	Assessment of compliance with the specific programme recommendations, with appropriate monitoring and support suited to the characteristics of patients and their disorder.
	-Supervision	Regular reminders and visits to a professional specialist in a personal and intimate context, which allows patients to converse in confidence.
	-Pharmaceutical	Managing of the antidepressant drugs, of possible improvement and their side effects.
	Cognitive habits	
	-Agency	Determination, personal effort when carrying out the program instructions, search for security and self-confidence.
	-Ruminations	Negative thoughts that hinder the implementation of the habits proposed in the programme.
	-Avoidance	Evasion of responsibilities via distracting activities on a cognitive level.
	-Restructurings	Modification of the values and ways of thinking in the sense of facilitating potential inclusion of the programme recommendations within the activities of daily life.
	-Guilt	Feelings of guilt derived from the failure to comply with the recommendations.
	Behavioural habits	
	-Activation	Mobilization of resources towards the carrying out of activities, breaking with inactivity and attempting to achieve behavioural objectives, little by little, generalized to all spheres.
	-Empowerment	To enable patients to lead their recovery.
	-Time pressure	Difficulties for giving instructions regarding change of habits in a short consultation.
	-Habit control	Personal ability to implement habit requirements and recommendations.
	-Opportunities	Socio-economic capacity to acquire certain foods, to follow certain instructions, and to carry out physical and pleasant activities.
Transversal aspects	Social support	
	-Group format of intervention	Social support coming from the group format of intervention, which helps to realize one is not alone when facing difficulties, in order to cope with depression and change habits.
	-Contact with others	People in the patient’s close social context – apart from family members and friends – that provide face-to-face contact and the opportunity for the acquisition of recommendations.
	Objectives	
	-Adjustment	Fit of the programme to the situation of each patient, with special interest in the prevention of recurrences once the patient has improved depression.
	-Intervention level	Special affinity of the programme with a level of individual intervention integrated in healthcare systems, although with possible connections with the media and educational systems.

## Discussion

According to the results of this study, certain personal, programmatic and transversal properties affect the general objective of modifying dietary and hygiene behaviours. These characteristics, which may act as barriers or facilitators, are all interrelated.

The properties particularly emphasized for modifying dietary and hygiene behaviours in patients suffering from depression include the need to conduct a simple and motivating intervention at the appropriate moment when the patient is receptive and capable of overcoming apathy. For this, the patient must receive the support and supervision of a healthcare professional. Thus, PC may be an appropriate level at which to implement lifestyle modification programmes in individuals suffering from depression, since the conditions for effective change can be provided by patient-centred care that is accessible, comprehensive and ongoing [28]. The intervention might be planned between a general practitioner/nurse and the patient, using a motivational interview, because health professionals are trained in the field of changing habits, and a tailored intervention can be set up [48, 49] based on their knowledge of the patient. Moreover, they are in a position to motivate and enable, assess patient evolution and provide constant feedback [50, 51].

The main barriers faced by patients are of a psychological nature and are directly related to the symptoms of depression: apathy, feelings of guilt or incompetence, intrusive thoughts, cognitive avoidance and the need for cognitive restructuring [3, 6]. These barriers reduce motivation and hinder compliance, which is why interventions must be appropriate and timely, and should take into account the course of the disorder [52]. Time pressures in medical consultations [53], the possible inability to implement recommendations, and more particularly, not having the purchasing power to implement and maintain healthy habits are also perceived barriers to changing habits by professionals, who highlight the importance of driving modifications from a behavioural point of view. As expressed by some participants, by way of example, a healthy diet is more expensive than an unhealthy one. In fact, several studies conducted on the general population highlight a relationship between affordable or lower prices and the consumption of fruits and vegetables, and the resulting impact on body weight [54–57]. This relationship is also evidenced in the opposite direction, i.e. a tax on certain types of food and drinks reduce their consumption [54–57]. Furthermore, it has been demonstrated that specific changes in diet are maintained while there is purchasing power [55], so this is a particularly important factor when implementing an intervention for the modification of dietary and hygiene behaviours.

On the other hand, facilitators are factors that enable programme adherence, such as the simplicity of recommendations, which do not require instrumentation and are largely dependent on only the patients themselves, the tailoring of recommendations to their situation and the importance of motivating patients. These factors are consistent with several studies [24, 36] that highlight the importance of giving simple, progressive and tailored messages depending on the evolution of the disorder, and frequent repetition [58]. Behaviour modification is a process that usually requires successive approaches to learning, and it is important for patients to play a proactive role in change. The new experiences must be beneficial but also rewarding, since this increases the probability of repetition [59]. Supervision and assessment of compliance have been considered facilitators by patients and health professionals, respectively. ICTs (mobile apps, software, etc.) are an option to improve monitoring or its intensity, and they may be able to serve as support and complementary tools for both patients and health professionals in this regard [36]. Another important facilitator category is the perceived social support provided by the social network, or even better, that provided by the programme itself, as affirmed by patients. The social support provided by family, friends, associations, groups, etc. helps to create an appropriate environment in which patients feel capable of changing their habits and behaviours [36]. Social support has been defined as a protective factor against depression [60, 61], but it is modulated by the subjects’ perceptions of the support that they receive; and for certain mental illnesses, it is known that depressed patients perceive less social support than they in fact have [62] . Thus, as has been observed by patients, professionals should try to create an intimate, trusting atmosphere in which patients feel entirely supported.

As discussed, general agreement exists regarding the idea that PC is the optimum level at which to implement lifestyle programmes for the modification of dietary and hygiene behaviours with depressive patients. However, other studies reveal that excessive workloads and time constraints to which health professionals are subjected make it difficult to implement these types of interventions in real practice, and continuous implementation is still sub-optimal [63, 64]. Educational, legislative and fiscal measures regarding food prices, environmental planning, and communication and marketing campaigns are important factors that have been highlighted by healthcare professionals to help assure the success of the intervention. This idea is consistent with results from other studies: fiscal measures regarding food prices help to regulate consumption [54–57] and marketing campaigns and audio-visual messages broadcast on television, in particular, have demonstrated their effectiveness in changing habits at a community level [65] through the use of emotional drivers to encourage behaviour change [66], such as affiliation, the presentation of aversive stimuli, etc. Nevertheless, at this time the role of the education system is still in question given the inconsistent results from several studies [67].

This study has certain limitations, mainly arising from the complexity of establishing a theoretical framework and taxonomy for changing lifestyles [68]. On the contrary, the main strength of this study is the triangulation of information related to the techniques of individual and group interviews, including the complementarity of patients and healthcare professionals, and therefore providing result with greater perspective. As for the generalizability of the results, the selection criteria for participants were defined with pragmatic intention; thus they are wide and relatively unrestrictive. This increases the heterogeneity of the sample and brings it closer to the real population of depressed patients served by PC professionals, while also allowing for the collection of a wide range of opinions.

## Conclusions

The implementation of intervention programmes that combine dietary and hygiene factors in patients with depression is complex, given the nature of the disorder itself and that of some of its main symptoms, such as apathy, the feeling of guilt, or ruminations on incompetence*.* However, key points exist for the success of the intervention, such as the simplicity of the guidelines, the tailoring of the intervention through motivational interviewing, and the prolonged and intense monitoring throughout the different stages of the disorder and the provision of adequate feedback. Taking into account its own limitations (e.g. time pressure), PC could be an appropriate level at which to implement these interventions, although educational, legislative and fiscal measures, environmental planning, and communication and marketing campaigns may be important factors that should also be considered.

## Abbreviations

ICT: Information and communications technology
PC: Primary Care
